# Annotations

**Published:** 1903-03-07

**Authors:** 


### Annotations.

### Epsom College.

Epsom College, the Royal Medical Benevolent College, is entitled to receive the support of every member of the community. It is the great benevolent institution of the medical profession, and every one from the King to the humblest citizen is enormously indebted to that profession. The fact that the Prince of Wales will preside at the fiftieth biennial dinner on July 6th next, when he has expressed a wish to receive at least £10,000 to free the College from all financial embarrassment, should produce not only £10,000, but more than that sum. It has been the privilege of the medical profession, in these realms, to give an amount of free professional service to the sick, through the hospitals, for centuries, which represents in the aggregate a monetary value, so large, as to be almost overwhelming to the mind of the thoughtful. The Prince of Wales's presence at this biennial dinner of Epsom College, in the year following the recovery of King Edward VII. from mortal illness and his coronation, should be welcomed, therefore, by every class of the community. Indeed, to the majority of individual citizens it offers a long-sought opportunity, when each of the healthy may properly by their gifts sent to the heir-apparent, express to all members of the medical profession sincere acknowledgments for the great eleemosynary service rendered by the profession to the whole community for centuries past. The administration, the management, the system of teaching, the discipline, the upkeep, all the arrangements of every kind connected with Epsom College inspire confidence. The most captious then can find no loophole or excuse whereby the most parsimonious of citizens can reasonably decline to give something towards the £10,000 now urgently needed. We look forward, therefore, with confidence to July 6th next, memorable as it will be by the presence of the Prince of Wales in the chair, and interesting, too, to the whole nation as the anniversary of the Prince's wedding day. It follows, if the knowledge of the simple facts here stated can be driven into the minds of the people, that the Biennial Dinner of Epsom College, 1903, may become a landmark in history through the generous recognition of a noble profession by the spontaneous gifts of a great people.

### Consumption and Charity.

While the world at large is being asked for funds for a tuberculosis sanatorium, a resident of Wimbledon has set to work to exploit the charitable public on his own account. Gaunt and bleak-looking, with a black respirator over his mouth and a few bootlaces in his hand, he sits in the street bearing on his bosom a placard with the word "Consumptive" printed in large letters upon it. Whether or not the man is really consumptive or is possessed of one of those convenient constitutions which make their possessor "blind" or "deaf and dumb" as the fashion in affliction and sympathy goes, the present writer cannot say, having in this instance imitated rather the Levite than the good Samaritan. But the sight struck him as needlessly unpleasant and even unwholesome. If the man was trying in an economic way to take the open-air cure, he would have been better up on Wimbledon Common, where the wind blows fresh and free, than in a comparatively narrow and busy street, and he would have been better without the probably dirty and microbe-laden respirator. But then he could not have sold his bootlaces or got coppers from kindly passers by without the formality of selling them, and the placard showed that he was making a business asset out of his disease. While the infectiousness of phthisis may be taken as proved, it is not so contagious that all who passed the unfortunate man, or even stopped to give him alms, were likely to contract it. It may therefore be that one's objection to seeing such a figure in our streets is only a sentimental one. Still, this fashion of displaying disease or infirmity in the public streets is one which the British public has long disliked, and which, if tolerated, will soon open the door to imposture on the one hand, and the offensive display of loathsome deformities on the other. If one man parades his tuberculosis why should not another wear a board with "cancer" writ large upon it, or any other ailment which will win sympathy and pence? Let us do our best to cure disease, but not to encourage sufferers to make a livelihood out of it.

### Hypnotism.

Hypnotism has now been allowed a recognised place in experimental psychology, and is admitted under necessary precautions as a legitimate and rational psycho-therapeutic agent. Much uncertainty, however, still remains as to its essential nature, and consequently in its application we are compelled to act in a manner empiric rather than follow a course clearly defined as scientific. Serious students of the subject will welcome the luminous and suggestive study presented in the epoc-making work of the late Mr. Frederic W. H. Myers on "Human Personality," recently published. Mr. Myers has sought to show that hypnotism is to be considered an experimental development of the sleeping phase of personality and that its effects resolve themselves into suggestion and self-suggestion. The main achievements of hypnotism seem to imply an increased subliminal vitalisation of the organism. Mr. Myers would define suggestion as a successful appeal to the subliminal self. Indeed, close analysis of "suggestibility" indicates that it signifies an increased internal responsiveness of the organism. This is what the physician constantly endeavours to arouse by the use of various nervous stimuli massive or specialised, and of the former, drugs afford a form which fortunately is often effective. The unpracticability of framing a physiological scheme of hypnotic results necessitates our falling back on psychological considerations and accepting inhibition and dynamogeny as a convenient contrast of conceptions. Mr. Myers' remarkable work supplies material which no serious student of hypnotism can afford to neglect, and which should receive the critical consideration of every physician.

